# Endoscopic removal of a massive trichobezoar in a pediatric patient by using a variceal ligator cap: A case report and literature review

**DOI:** 10.3389/fmed.2022.1020648

**Published:** 2022-11-04

**Authors:** Dan Lu, Björn Berglund, Yi Xia, Ajay Jain, Qing Gu, Feng Ji

**Affiliations:** ^1^Department of Endoscopy Center, The First Affiliated Hospital, College of Medicine, Zhejiang University, Hangzhou, China; ^2^Department of Biomedical and Clinical Sciences, Linköping University, Linköping, Sweden; ^3^Department of Gastroenterology, The First Affiliated Hospital, College of Medicine, Zhejiang University, Hangzhou, China; ^4^Meridian Medical Group, Indiana University Health Methodist Hospital, Indianapolis, IN, United States

**Keywords:** endoscopy, removal, trichobezoar, pediatric, case report

## Abstract

A trichobezoar is commonly formed in the gastrointestinal tract by ingestion of an individual’s own hair. A trichobezoar formed by hair and artificial materials constitutes a rare etiology scarcely reported in the current literature. A mixture with hair-like synthetic fibers not only increases the risk for trichobezoar formation but also makes it more difficult for endoscopic removal. Herein, we report on a case in which a trichobezoar, caused by the consumption of human hair and synthetic yarn, was successfully removed endoscopically with a variceal ligator cap without further complications for the patient. This case report aims to raise awareness among endoscopists that using a variceal ligator cap may be a suitable option in the management of large trichobezoars containing synthetic fibers.

## Introduction

A trichobezoar is a collection of ingested hair, usually the individual’s own hair, that accumulates in the gastrointestinal tract, commonly located in the stomach (1). Trichobezoars may lead to life-threatening complications, such as intestinal obstruction, gastrointestinal hemorrhage, and multiple perforations (2). Trichobezoars have traditionally been removed by laparotomy or laparoscopic-assisted laparotomy. Even though the endoscopic method has become the preferred treatment for trichobezoars and the success rate of using the method has increased from 5% to 31% during the last 10 years, endoscopic removal of large trichobezoar formed by hair and synthetic materials is still challenging for endoscopists (3).

Trichobezoars are mostly encountered in female pediatric patients with psychiatric disorders (4–6). In rarely reported cases, patients ingest not only their own hair, but also hair-like materials, such as artificial hair extensions or dolls’ hair (3, 7–9). When the trichobezoar is mixed with hair-like synthetic fibers, matted synthetic hair increases the risk for trichobezoar formation (8). Furthermore, these synthetic fibers can get twisted with the hair, making it more difficult for endoscopic removal.

There is currently no consensus regarding which method to apply for the removal of large trichobezoars mixed with large amounts of synthetic fibers. However, the commonly applied electrical devices are not suitable, as the synthetic fibers are composed of polyester and nylon (3, 8), which are hazardous when burnt (3, 10). According to a previous case, endoscopic removal of trichobezoars mixed with synthetics by using electrically based endoscopic devices causes severe procedural and/or post-procedural perforations in the small bowels as the synthetic materials are burnt (3). Herein, we report on a case in which a trichobezoar, caused by the consumption of human hair and synthetic yarn, was successfully removed endoscopically with a variceal ligator cap.

## Case presentation

An 11-year-old girl presented at a local hospital with a 6-month history of recurrent upper abdominal pain. An upper gastrointestinal contrast swallow study revealed the presence of a large intragastric filling defect, and abdominal ultrasound revealed the presence of a large gastric echogenic mass. The patient’s caretakers refused further evaluation by gastroscopy. The patient visited the local hospital a second time after her abdominal pain had worsened for 2 days. Endoscopic evaluation and the esophagogastroduodenoscopy showed a gastric foreign body (trichobezoar), a duodenal foreign body (consisting of a few strands of hair), and a gastric angle ulcer. Surgical removal was recommended. However, the patient’s caretakers refused surgical treatment and the patient was transferred to our tertiary health care hospital to seek further management options.

The patient started ingesting her own hair and yarn from her own clothes about 2 years earlier without telling her parents and the diagnosis of trichotillomania or trichophagia had not been made before. Otherwise, her medical and family history was unremarkable. Her body temperature was 37.0°C and her vital signs were stable on admission. The patient presented with the chief complaint of abdominal pain with an associated loss of appetite. Abdominal examination was positive for upper abdominal tenderness, but otherwise was without remarks. Neurological, cardiovascular, and respiratory exams were unremarkable. The C-reactive protein (CRP) level was 47.45 mg/L (reference range 0.0–8.0 mg/L). Total cholesterol was 2.89 mmol/L (reference range 3.14–5.86 mmol/L). All other laboratory results were normal. More detailed laboratory data is in the Supplementary Table 1.

**FIGURE 1:**
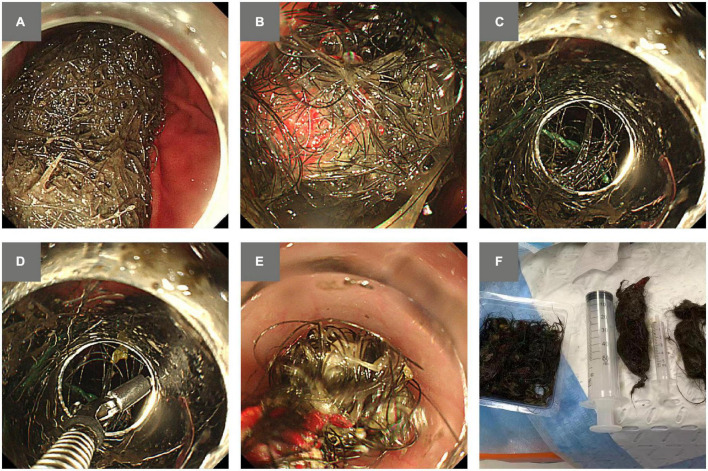
Images of the trichobezoar stuck in the stomach and attempts of endoscopic removal. **(A)** A trichobezoar was occupying the lumen of the stomach. **(B)** The trichobezoar contained synthetic yarn. **(C)** A variceal ligator cap was attached to provide more flexible maneuvering space for positioning the hairball in the cap. **(D)** Grasping forceps were applied to loosen the tightly intertwined forces. **(E)** Piecemeal removal of the hair strands and yarn by using grasping forceps. **(F)** Complete removal of the trichobezoar.

Considering a less invasive procedure than surgical intervention, a subsequent endoscopic attempt of trichobezoar removal was arranged for the patient. The procedure was performed under general anesthesia with endotracheal intubation. An endoscope (GIF-Q260 J, Olympus Corp, Tokyo, Japan) attached with a 12.4 mm transparent cap (Olympus Corp) was used for the procedure. A 10 cm × 10 cm mass consisting of hair mixed with yarn was found, occupying the lumen of the stomach (Figures 1A,B). Considering the risk of perforation (3), caused by surgical knives and APC when removing trichobezoars containing synthetic fibers, the endoscopist elected to avoid using electrocautery. As the trichobezoar was densely impacted, initial attempts at removal by using endoscopic scissors and a polypectomy snare were unsuccessful. Instead, the gastroscope was withdrawn and loaded with a variceal ligator cap (Six-Shooters S MBL-6; Wilson-Cook Medical, Winston-Salem, NC, USA). The variceal ligator cap enabled a more flexible maneuvering space (Figure 1C). It was used to position the hair strands into it when the grasping forceps was repeatedly pulling the densely impacted hairball back and forth in order to loosen it (Figure 1D). After several hours of attempts, the hairball became less firm. The grasping forceps and the polypectomy snare was able to grasp the hair strands into the variceal ligator cap and retreat outside the stomach alternatively. Cautiously, the hair strands and yarn were removed piecemeal (Figure 1E). The hairball and yarn were completely removed in about 20 passes (Figure 1F). When the endoscope was inserted again, three mucosal abrasions were spotted at the entrance of the esophagus, with no evidence of tears, and norepinephrine normal saline diluent was sprayed to prevent bleeding. The endoscopic procedure took in total about 5.5 h.

The patient was in postoperative anesthesia status, with a body temperature of 37.3°C, heart rate of 70/min, and blood pressure of 116/96 mmHg. She was transferred to the intensive care unit for further monitoring. Cefuroxime (0.75 mg every 8 h) was administered as infection prophylaxis, pantoprazole was administered to inhibit gastric acid, and an intravenous drip was administered to maintain electrolyte balance and for nutritional support. On the first night post-procedure, laboratory results showed: neutrophils 87.6% (reference range 50.0–70.0%), and CRP 31.73 mg/L (reference range 0.0–8.0 mg/L). On post-procedure day 1, the patient was transferred to the Department of Gastroenterology to continue the supportive treatment with pantoprazole inhibiting gastric acid secretion and nutrition. Blood testing of the patient showed: neutrophils 69.5% (reference range 50.0–70.0%), CRP 29.83 mg/L (reference range 0–8.0 mg/L). More detailed laboratory data is presented in Supplementary Table 1. The patient recovered without procedural complications. The patient underwent further psychiatric treatment after she was discharged on post-procedure day 3.

## Discussion

Bezoars are classified into different types depending on their composition.

Unlike other bezoars, trichobezoars are associated with psychiatric disorders, typically trichophagia and trichotillomania (an urge to pull out and consume hair), which are most commonly found among young women. It has been estimated that only 5–10% of patients who suffer from trichotillomania also engage in trichophagia. The underlying mechanisms for trichotillomania and trichophagia are still not yet clear, but the behaviors of pulling the hair out, and consuming it are associated with a sense of gratification and negative emotion relief. Behavioral therapy or response prevention initiated in early life has shown to have a decreased risk of relapse as compared to adults seeking treatment (11, 12).

Two common types of bezoars are phytobezoars, a concretion of indigestible fibers derived from ingested vegetables and fruits (13), and trichobezoars, which are caused by trichophagia (14). Phytobezoars are the more common of these two, however, trichobezoars are the most difficult type of bezoar to treat. Phytobezoars may be softened by the consumption of carbonated soft drinks or enzymatic dissolution, followed by endoscopic fragmentation by using endoscopy tools whereas the tightly interlaced strands of hair of trichobezoars appear to be less susceptible to chemical softening or to endoscopic fragmentation and eventual removal (3, 14). For example, Iwamuro et al. reported on attempting dissolution of the trichobezoar with carbonated soft drinks, resulting in failed fragmentation (15). Wang et al. reported that needle-knife is effective for removing trichobezoars, whereas diospyrobezoars could be treated with mechanical lithotripter (16). Therefore, trichobezoars typically require removal by gastrotomy, with broad-spectrum antibiotic coverage (17).

Endoscopy-based methods have become the alternative choice for trichobezoar removal since Van Gossum et al. reported on the first case in which a trichobezoar could not be removed despite using laser, water jets and extracorporeal shock-wave lithotripsy (18). However, there has been an increase in the success rate of endoscopic removal attempts of trichobezoars in recent years. This may be attributable to an increasing number of available endoscopic methods as well as an increased willingness by the endoscopist to use the endoscopic approach to removal (3). Wang et al. began to use a modified needle knife to fragment and remove a 10 cm long trichobezoar (16). One review of trichobezoar removal found 16 successful vs. 36 cases of unsuccessful endoscopic removal attempts, estimating a success rate of 30.7% (3), whereas an exhaustive review, published by Gorter et al. in 2010, reported a 5% success rate of trichobezoar removal by endoscopic methods (19).

In our study, database PubMed was searched from its beginning until September 25, 2022. The search terms included “trichobezoar” OR “trichophytobezoar” AND “endoscopy” OR “endoscopic.” Titles and abstracts of the 107 results were evaluated, and the publications that qualified were read in full. This resulted in the inclusion of 18 articles of 19 cases, on successful endoscopic removal of trichobezoars, as 1 article was a series of 2 cases report. The remaining 89 articles reported on trichobezoars removed by surgical interventions, like laparotomy, gastrotomy, and enterotomy. Patient demographics, extension, location, and size of the trichobezoars and used endoscopic devices for trichobezoar removal and the duration of the procedures are outlined in Table 1.

**TABLE 1:** Successful endoscopic retrieval of trichobezoars in the literature.

No.	Authors	Patient’s age and sex	Extension	Location	Size	Endoscopic removal devices	Duration	Outcome
1	Soehendra (23)	17/F	Hair	Stomach	15 cm × 7 cm.	Flexible outer tube	Three sessions of 2–3 h each	Successful removal
2	Timoshchenko et al. (24)	N/A 2 cases	N/A	Stomach	N/A	N/A	N/A	Successful removal
3	Wai et al. (25)	56/M	Hair	Stomach	N/A	An endoscopic snare	N/A	Successful removal
4	Konuma et al. (22)	9/F	Hair	Stomach	1.8 cm × 3.2 cm × 34 cm	Grasper 5-prolongs and retrieval net	15 min	Successful removal
5	Esmaili et al. (26)	17/F	Hair	Stomach	N/A	By esophagoscope	N/A	Successful removal
6	Aybar et al. (4)	5/F	Hair	Stomach extending through the duodenal bulb	8 cm × 7 cm.	Hot biopsy forceps and a snare	3 h	Complete removal with mucosal abrasions and small superficial mucosal burns in the greater curvature
7	Renji et al. (27)	12/F	Hair	Oesophagus	N/A	Removed with the PEG tube	N/A	Successful removal
8	Iwamuro et al. (15)	10/F	Hair	Stomach	N/A	Electrosurgical knives after APC, polypectomy snare, and Coca-Cola administration failed	N/A	Successful removal
9	Kao et al. (28)	5/F	Own hair, doll hair, stuffed animal hair	Stomach	4 cm with dumbbell shape	Magill forceps	N/A	Complete removal with moderate supraglottic edema
10	Benatta (5)	6/F	Hair	Stomach with a few extension through the pylorus	8 cm × 4 cm	Polypectomy snare and APC	50 min	Completely removal with mucosal polypoid ulceration in the greater curvature and duodenal linear laceration
11	Amjad et al. (20)	37/F	Hair	Stomach	N/A	Endoscopic scissors, Rat Tooth forceps, Tri-Prong forceps, and a Roth net.	N/A	Successful removal
12	Zhao et al. (29)	12/F	Hair and vegetable fibers	Stomach with a few extension through the pylorus	10.5 cm × 3.5 cm	Polypectomy snare, APC, grasping forceps, biopsy forceps, sodium bicarbonate solution, and scissors	N/A Twice endoscopic treatment 5 days apart	Successful removal
13	Chun et al. (30)	4/F	Hair	Stomach and jejunum	N/A	Electrosurgical knife (IT knife)and snare through a single-balloon enteroscopy	N/A	Successful removal
14	Niţã et al. (3)	9/F	Hair and Doll’s hair	Stomach and duodenum	25 cm × 30 cm	Argon plasma coagulation (APC) and snare electrocautery	3 h of 80% mass removal	Complete removal with 18 small bowel perforations
15	Baidwan et al. (11)	11 month/M	Hair, nail	Stomach	10 cm × 3 cm	Through his G-tube	N/A	Successful removal
16	Baek et al. (2)	22/F	Hair	Stomach	6 cm × 15 cm	An electrosurgical knife (IT knife) after grasping forceps, APC and a polypectomy snare failed	2 h	Successful removal
17	Wang et al. (31)	9/F	Hair	Stomach	11.6 cm × 5.6 cm × 4.9 cm	Forceps, snare, and an overtube channel	N/A	Successful removal
18	Wang et al. (32)	14/F	Hair	Stomach	12 cm × 6 cm	Endoscopic coagulation cutting by a polypectomy snare and a Hook knife after grasping forceps and Dormier-type stone retrieval basket failed	1 h	Completely removal with multiple gastric ulcers detected

Among these 19 endoscopic retrieval cases, electrical-based endoscopic devices were applied in 9 cases. In 2 case reports, endoscopic devices were not available. Other modalities included removal through a G-tube (1 case) and PEG tube (1 case). Non-electrical based endoscopic devices in the remaining six cases (overtubes in 2 cases, a combination of forceps, grasper, retrieval net, and scissors in 3 cases, by esophagoscope in 1 case). In the 9 cases in which electrical-based approaches to removal were used, electrosurgical knives were succesfully applied in 3 cases (IT knives were used in 2 cases and a Hook knife in 1 case).

In some cases, trichobezoars are formed not only by ingestion of the individual’s own hair, but also by ingestion of hair-like synthetics, such as artificial hair extensions or dolls’ hair (3, 7–9). Hair-like synthetics is composed of synthetic fibers, such as polypropylene, alkyd resin, rayon, polyester, nylon, and acrylic, which can be hazardous when burnt. Synthetic yarn is composed of similar components.

When treating trichobezoars, electrical and non-electrical devices could be alternative endoscopic tools. But endoscopic removal of large trichobezoars mixed with synthetic fibers by using commonly applied electrical endoscopic devices could possibly lead to the formation of noxious fumes. Formed hydrogen chloride may travel through the intestine, and following the combination with water and gastric juices, could cause mucosal damage and lead to perforation of the gastrointestinal wall (3). Niţã et al. presented a case, in which a trichobezoar mixed with human hair and synthetic hair of dolls was fragmented by using APC and snare electrocautery, but 18 small bowel perforations, including the most proximal one located at 5 cm from duodeno-jejunal flexure and a 1 cm posterior gastric perforation into the lesser sac, were detected in later esophagogastroduodenoscopy. Gastrotomy, jejunostomy, and small bowel resection were performed due to severe complications caused by the application of electrical devices (3).

Furthermore, the components of synthetic materials are resistant to acids, weak alkalis, and organic solvents, which results in the strands persisting in the stomach, increasing the risk of gastrointestinal obstruction and bleeding. Moreover, artificial materials can be tangled with natural hair and make it even more difficult for endoscopic removal (8), as in our case, in which initial attempts to remove the trichobezoar with scissors were unsuccessful. However, Amjad et al. presented a case in which endoscopic scissors were used to fragment a large trichobezoar into smaller pieces before successful endoscopic retrieval (20). In our case, hair was twisted with artificial yarn to change the physical properties of the trichobezoar, making it more challenging to cut off.

A few cases in which succesful removal of trichobezoars in one piece without fragmentation was performed, have previously been reported on. Saeed et al. presented a case, in which a bezoar was removed by grasping with a “pelican-type” forceps and partly engaged into an overtube. The 12-cm long trichobezoar was removed together with the overtube as a single unit, without fragmentation (21). Konuma et al. successfully retrieved a gastric trichobezoar without fragmentation by using only a grasper and a net. This could be accomplished due to the favorable, longitudinal shape (34 cm long) and diameter (1.8 cm) of the trichobezoar (22). Both masses in these two cases were by chance oblong, which may have allowed for complete removal of the bezoar without fragmentation. However, in the majority of cases, endoscopic methods fail to remove the trichobezoar as a whole (9).

In this case report, the variceal ligator cap provided a safe space for flexible maneuvering and enabled the endoscopist to eventually loosen the core of the trichobezoar by repeated pulling by using endoscopic grasping forceps. The potential for performing truly scar-less, safer procedures, as well as with lower rates of complications, is appealing to both physicians and patients. The caregiver of the patient in our case refused initial surgical treatment and accepted later endoscopic treatment. It is more acceptable to remove trichobezoars *via* a natural orifice than through routine laparoscopic or surgical operations. Furthermore, endoscopic removal is more cosmetically advantageous as it leaves no scars.

A disadvantage of the method is that it is time-consuming and laborious, which poses a great challenge to the endurance and patience of the endoscopist.

However, by using the method, complications such as large-scale perforation caused by cauterization with an electronic knife and APC can be avoided. Endoscopic removal of trichobezoars formed by human hair and synthetic fibers by using a variceal ligator cap is minimally invasive. In conclusion, non-electrical endoscopic devices, like a variceal ligator cap, may be suitable options in the management of trichobezoars in selected cases.

## Data availability statement

The original contributions presented in this study are included in the article/Supplementary material, further inquiries can be directed to the corresponding authors.

## Ethics statement

Ethical permission for this study was approved by the Clinical Research Ethics Committee of the First Affiliated Hospital, College of Medicine, Zhejiang University (IIT20220284A). Written informed consent to participate in this study was provided by the participants’ legal guardian/next of kin. Written informed consent was obtained from the individual(s), and minor(s)’ legal guardian/next of kin, for the publication of any potentially identifiable images or data included in this article.

## Author contributions

DL and FJ conceived of the study. DL, YX, QG, and FJ collected and analyzed the clinical history and data. DL, BB, and AJ drafted and critically revised the manuscript. All authors contributed to the article and approved the submitted version.
