# Supplementary material for: Endoscopic removal of a massive trichobezoar in a pediatric patient by using a variceal ligator cap: A case report and literature review
Source: Front Med (Lausanne). 2022 Nov 4;9:1020648. doi: 10.3389/fmed.2022.1020648 (PMC9673752; doi:10.3389/fmed.2022.1020648)
Supplement: Supplementary file 1 [file Table_1.pdf]

Supplementary Table 1. Laboratory Data

Variables	Reference range	On admission	On the first night post-procedure	On post-procedure day 1
Blood				
Hemoglobin (g/L)	110-155	128	122	114
WBC (10E/L)	4.0-12.0	5.83	7.13	8.78
Neutrophils (%)	50-70	58.6	87.60%	69.50%
Lymphocytes (%)	20-40	25.6	8.60%	19.60%
CRP (mg/L)	0.0–8.0	47.45	31.73	29.83
Albumin (g/L)	38-54	41.1	37.7	34.9
Total cholesterol (mmol/L)	3.14-5.86	2.89		
